# Integrating citizen science and environmental DNA metabarcoding to study biodiversity of groundwater amphipods in Switzerland

**DOI:** 10.1038/s41598-023-44908-8

**Published:** 2023-10-23

**Authors:** Marjorie Couton, Angela Studer, Samuel Hürlemann, Nadine Locher, Mara Knüsel, Roman Alther, Florian Altermatt

**Affiliations:** 1 Department of Aquatic Ecology, Eawag: Swiss Federal Institute of Aquatic Science and Technology, Überlandstrasse 133, 8600 Dübendorf, Switzerland; 2 Department of Evolutionary Biology and Environmental Studies, University of Zurich, Winterthurerstrasse 190, 8057 Zürich, Switzerland

**Keywords:** Biodiversity, Freshwater ecology, Molecular ecology

## Abstract

Groundwater is the physically largest freshwater ecosystem, yet one of the least explored habitats on earth, both because of accessing difficulties and the scarcity of the organisms inhabiting it. Here, we demonstrate how a two-fold approach provides complementary information on the occurrence and diversity of groundwater amphipods. Firstly, we used a citizen science approach in collaboration with municipal water providers who sampled groundwater organisms in their spring catchment boxes over multiple weeks, followed by DNA barcoding. Secondly, we collected four 10 L water samples at each site, in one sampling event, for environmental DNA (eDNA) metabarcoding. We found that citizen science was very effective in describing the distribution and abundance of groundwater amphipods. Although the single time-point of eDNA sampling did not detect as many amphipods, it allowed the assessment of the entire groundwater community, including microorganisms. By combining both methods, we found different amphipod species co-occurring with distinct sequences from the eDNA-metabarcoding dataset, representing mainly micro-eukaryotic species. We also found a distinct correlation between the diversity of amphipods and the overall biodiversity of groundwater organisms detected by eDNA at each site. We thus suggest that these approaches can be used to get a better understanding of subterranean biodiversity.

## Introduction

Groundwater is the largest type of freshwater ecosystem on earth^1^. It harbors complex communities with organisms from various groups, from prokaryotes to macroinvertebrates, including chromists, protozoans, and fungi^2^. Amphipods are among the most abundant and diverse macroinvertebrate group in groundwater^3,4^, with more than 200 genera described, encompassing around 1000 species^5^. Among them, the genus *Niphargus* Schiödte, 1848 is the most species-rich genus of amphipods in the world^6^, encompassing 418 species described to date^7^, most of them living in subterranean habitats. Amphipods provide ecosystem services, such as particulate organic matter breakdown, maintenance of hydraulic conductivity via bioturbation, or the elimination of pathogenic microorganisms^8,9^. Their sensitivity to pollutants makes them good candidates for being used as bioindicators, as was already demonstrated for surface ecosystems (e.g.,^10,11^), but also in groundwater (e.g.,^12^). However, despite their large diversity and undeniable value, groundwater amphipods (and stygofauna in general) are still largely understudied.

As opposed to surface taxa, the number of newly described groundwater species is still steeply increasing^13,14^. This suggests that we are far from having reached the total number of existing species, which might even be higher than for surface ecosystems. Although Europe harbors the highest described and known groundwater diversity in the world, including many biodiversity hotspots (e.g., the Western Balkans or the Pyrenees^2,15^), our knowledge on stygofauna is still limited. This is due to three main factors. First, most subterranean habitats are inaccessible to humans, prohibiting a proper exploration of these environments as compared to surface ecosystems, an issue coined as the Racovitzan impediment by Ficetola, et al.^13^. Second, the density of organisms living in groundwater is low and many species exhibit short-range endemism^16^. Consequently, many species may only be found with high and adequate sampling efforts, which is particularly challenging as access to groundwater ecosystems is often very localized. To adequately record the stygofauna’s diversity and distribution at a given location, it is thus important to know the methods’ sensitivity to detect the target species^2,17^. Finally, many species are not yet formally described and/or lack distinct morphological criteria, creating challenges in identifying and cataloguing species in general, and leading to a largely unresolved groundwater taxonomy with numerous cryptic species (e.g.,^18^). To date no single method to sample and assess groundwater biodiversity is addressing and resolving these three problems successfully.

One promising approach to the study of groundwater organisms is the use of environmental DNA (eDNA), where organisms’ occurrence is derived through the presence of their DNA in water or soil samples^19–21^. Several studies have already investigated the potential of eDNA for groundwater^22^, either by using species-specific techniques to detect one or a few organisms (e.g.,^23,24^), or by applying a metabarcoding approach to get a description of whole communities (e.g.,^25–27^). The application of a molecular method is particularly adapted for studying the variety of groundwater cryptic species. Moreover, using water as a sampling medium is ideal to overcome the accessibility issue, as water can be pumped or captured directly where it naturally flows out of the ground. Several limitations, however, can hamper the use of eDNA in groundwater, such as the lack of reference sequences in public databases for taxonomic assignments in metabarcoding approaches^25,27^, or the lack of suitable primers for groundwater organisms^22^. Moreover, eDNA does not provide phenotypic information (e.g., sex, life-stage), and does not allow the description of new species, which generally depends on the availability of actual specimens for morphological description and designing a type specimen. Given that undescribed species are still numerous in subterranean environments, the necessity of actually sampling organisms is still high.

**Figure 1:**
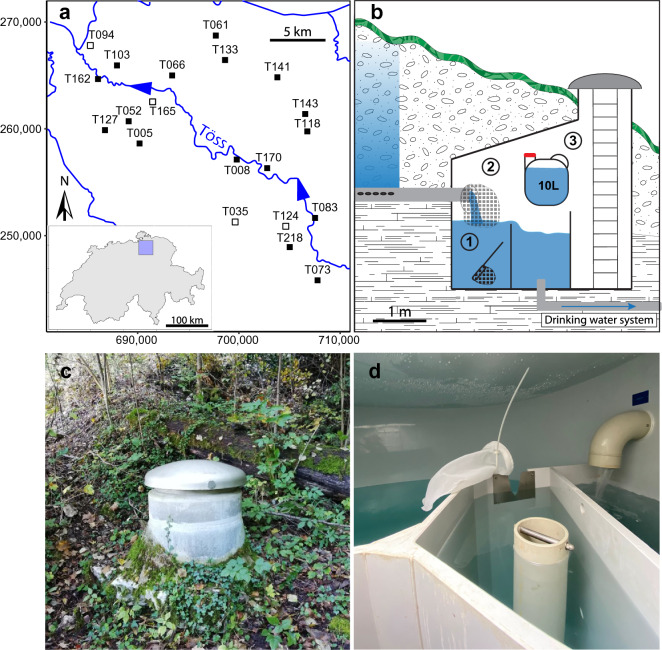
Sampled sites and depiction of the sampling protocol. (**a**) Map of the catchment area of the river Töss (North-Eastern Switzerland), displaying the 20 sampled sites. Filled squares indicate a site with amphipods, and empty squares represent sites where no amphipods could be identified. The coordinates used are from the Swiss LV03 system. (**b**) Sampling at each site was performed in a Spring Catchment Box (SCB) built to passively collect water from the aquifer. For the citizen science approach, water providers collected organisms present in the overflow basin with an aquarium net (1), then they fixed a filter net to their outflow pipe and checked it every week to collect captured organisms (2). Water for eDNA sampling was collected directly from the outflow pipe in a plastic container and brought back to the lab for filtration (3). The scale is only an approximate indication, as every SCB is different. (**c**) and (**d**) are pictures of an SCB from the outside or the inside respectively.

Here, we address the three main aforementioned issues for studying groundwater organisms by using a combination of two methods, one based on sampling actual specimens using a citizen science approach followed by DNA barcoding, the other based on water sampling and subsequent eDNA metabarcoding. Amphipod individuals and groundwater samples were collected in 20 spring catchment boxes (SCBs; Fig. 1) in North-Eastern Switzerland, in a collaboration with communal water providers. We evaluated the effectiveness of both methods to retrieve information on the diversity and distribution of groundwater amphipods. Then, we assessed the potential of combining both approaches to gain insights on the relationship between the identified amphipod species and the overall groundwater community.

## Results

### Citizen science

Within the 20 sites sampled by the water providers, a total of 272 individual amphipods were collected from 17 sites and sent to our lab (average sampling duration of 46 days, standard deviation [SD] = 23). Three sites, T035, T124, and T165, were devoid of amphipods (Fig. 1a), despite having been sampled for 69, 62, and 29 days respectively. At one site (T094), only one amphipod individual was collected, but could not be assigned to any species due to poor DNA quality. We successfully sequenced and assigned 254 of the collected individuals to a groundwater amphipod species (see Supplementary Table S1 online). We identified six different species, five belonging to the genus *Niphargus* (i.e., *Niphargus auerbachi* Schellenberg, 1934, *Niphargus fontanus* Spence Bate, 1859, *Niphargus puteanus* (Koch, 1836), *Niphargus thienemanni* Schellenberg, 1934, and *Niphargus tonywhitteni* Fišer, Alther, Zakšek, Borko, Fuchs & Altermatt, 2018) and one being *Crangonyx subterraneus* Spence Bate, 1859. All of these species were previously reported in Switzerland^14,28,29^. The most widespread species (*N. tonywhitteni,* 42 individuals, and *N. auerbachi*, 30 individuals) were detected in seven sites, whereas *N. thienemanni*, the least widespread (two individuals), was only found in one site (Table 1). All assignments were successful when comparing to our internal database with more than 99% identity. Only *C. subterraneus* could not be assigned based on local references and was assigned based on a comparison with GenBank nt database (between 94.2% and 96.5% identity; see Supplementary Table S1 online).

**Table 1:** Abundance (in number of individuals or number of reads) and frequency (in number of sites) of the six groundwater amphipod species detected with the citizen science and/or the eDNA metabarcoding approach.

Species	Citizen science		eDNA metabarcoding	
	Individuals	Sites	Reads	Sites
*Crangonyx subterraneus*	10	5	0	0
*Niphargus auerbachi*	30	7	23	1
*Niphargus fontanus*	79	5	412	2
*Niphargus puteanus*	91	2	254	1
*Niphargus thienemanni*	2	1	284	1
*Niphargus tonywhitteni*	42	7	76	3

### Metabarcoding

Sequencing of the 20 water sample replicates per site (5 tagged PCR replicates per filter, 4 filters per site) produced 13,584,846 reads with a mean of 139,678 (SD = 104,236) reads per index combination (i.e., filter replicate) and a mean of 558,713 (SD = 276,277) reads per site. The use of dada2 resulted in the production of 10,799 unique sequences called Amplicon Sequence Variants (ASVs), with only 4,917 remaining after index-jump correction and removal of potential PCR errors (5,657,618 reads). After pooling data from the 20 sampling replicates per site, each site exhibited a mean number of 282,881 reads (SD = 169,339) and a mean number of 574 ASVs (SD = 481; see Supplementary Table S2 online). T133 was the only site with only three filter replicates (15 total replicates) because the extraction of one of the filters did not work properly.

When comparing all ASVs against our lab-internal amphipod database (including the sequences produced by the citizen science approach), seven ASVs (encompassing 1,049 reads) were assigned to a groundwater amphipod species. In total, we identified five species, all from the genus *Niphargus* (i.e., *N. auerbachi*, *N. fontanus*, *N. puteanus*, *N. thienemanni*, and *N. tonywhitteni*). All assigned ASVs were identical (or nearly identical: 99.5% identity) to a reference in our database (see Supplementary Table S3 online).

### Comparison of the two methods

Overall, both methods detected the same five species from the genus *Niphargus*. *Crangonyx subterraneus* was only detected by the citizen science approach. However, since the primers were only tested on species from the genus *Niphargus*, the lack of detection of *C. subterraneus* might be the result of an amplification bias for this species.

**Figure 2:**
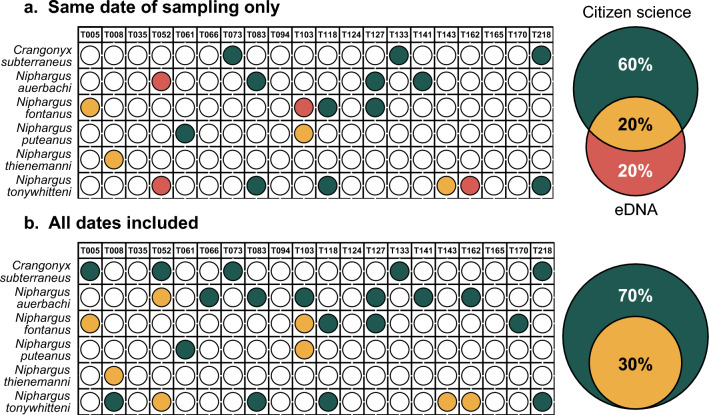
Amphipod detection efficiency of eDNA metabarcoding, after removing reads to account for PCR errors and index-jump, as compared to the citizen science approach. (**a**) comparison of the two approaches when considering only citizen science samples collected at the same date as eDNA sampling. Each colored dot represents a detection for a particular species (y-axis) at one site (x-axis). Green dots represent detections by citizen science only, red dots by eDNA metabarcoding only and yellow dots by both approaches. The Venn diagram on the right shows the proportion of detection for each or both methods. (**b**) comparison of the two approaches when considering all citizen science samples collected for this study.

When comparing the efficiency of both methods at each site, only 20% of the detections were congruent between approaches at the same sampling time (Figs. 2a and Supplementary Fig. S1a online). Additional detections were performed by both methods separately, with 20% of them being attributed to eDNA and 60% to citizen science. When including all the temporal replicates of the citizen science approach into the comparison, all detections previously attributed to eDNA only did match with the citizen science approach (Fig. 2b and Supplementary Fig. S1b online). In total, 30% of the detections are congruent between approaches and 70% are only attributed to citizen science.

**Figure 3:**
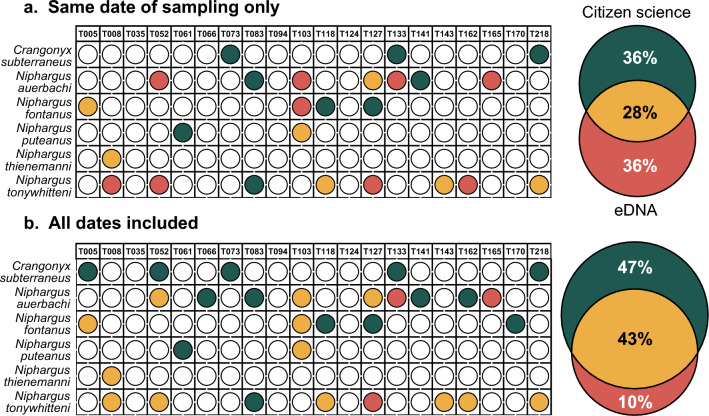
Amphipod detection efficiency of eDNA metabarcoding, before removing reads to account for PCR errors and index-jump, as compared to the citizen science approach. (**a**) comparison of the two approaches when considering only citizen science samples collected at the same date as eDNA sampling. Each colored dot represents a detection for a particular species (y-axis) at one site (x-axis). Green dots represent detections by citizen science only, red dots by eDNA metabarcoding only and yellow dots by both approaches. The Venn diagram on the right shows the proportion of detection for each or both methods. (**b**) comparison of the two approaches when considering all citizen science samples collected for this study.

Since amphipod species represent only a very small fraction of our eDNA metabarcoding dataset (0.001% of ASVs and 0.0002% of reads), we also compared the results of the citizen science approach to the eDNA data before correction to remove PCR errors and to account for index-jump. In this case, the number of detections with eDNA doubled (16 detections) and, although the congruence between the two approaches at the same time point remained in the same proportion (28%), the fraction of detections made solely by eDNA increased (36%; Fig. 3a). When considering all the sampling time points from the citizen science approach, the congruence increased (43%) and 10% of detections were solely made by eDNA (Fig. 3b). Importantly, however, some of the ASVs included in this study were present at a lower abundance in some samples than in negative controls for index-jump. This means that we cannot resolve if they are true detections or the result of the attribution of the wrong index during demultiplexing.

### Co-occurrences patterns between amphipods and ASVs

**Figure 4:**
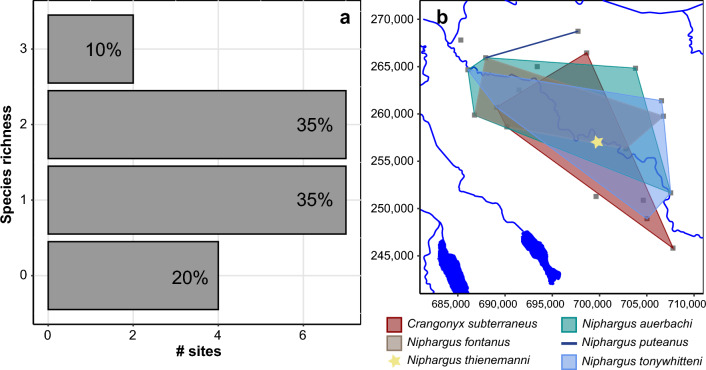
Distribution of amphipod species revealed by the citizen science approach. (**a**) barplot indicating the number of sites for which none, one, two or three species were found with the citizen science approach. The proportion over the total number of sampled sites is indicated at the top of each bar. (**b**) Polygons of the distribution of each amphipod species identified with citizen science over the sampled area. The line connects two sites and the star indicate presence at only one site. Each sampled site is represented by a grey square. The coordinates used on the axes are from the Swiss LV03 system.

**Figure 5:**
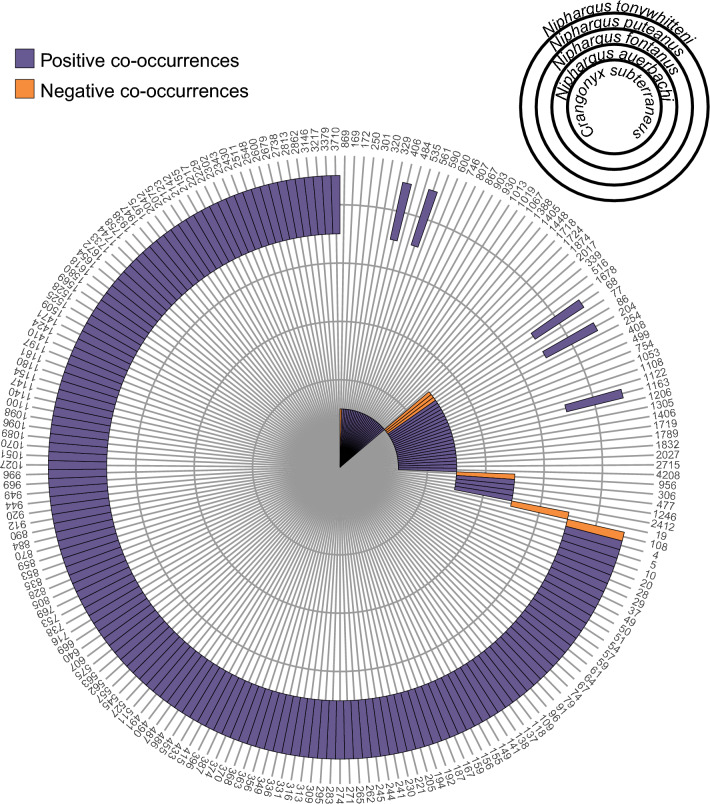
Co-occurrences between amphipod species identified with citizen science and ASVs from eDNA metabarcoding. Positive co-occurrences (one amphipod species occurring together with one ASV at more sites than would be expected by chance) are shown in purple, while negative co-occurrences (one amphipod species occurring together with one ASV at less sites than would be expected by chance) are shown in orange. Only significant co-occurrences are displayed (*p* > 0.05). Only ASVs with either a positive or negative co-occurrence with an amphipod species are presented in this figure (ASV number on the outer part of the circle). Each amphipod species corresponds to one concentric circle (see legend on the top right).

Although the citizen science approach identified six amphipod species overall, multiple of them were rarely found together in one site. Only one or two species were observed in the majority of sites (87.5% of the sites where amphipods are present; Fig. 4a). However, the distribution of the species detected at more than two sites covered most of the sampled area. Therefore, these species have greatly overlapping distributions in the Töss catchment (Fig. 4b). To better understand this lack of co-occurrence between amphipod species in our dataset, we compared the distribution of each amphipod species from the citizen science approach with the distribution of all ASVs produced with eDNA metabarcoding. The four amphipod species identified in more than two sites positively co-occurred with a total of 194 ASVs, and negatively co-occurred with seven ASVs (Fig. 5, Supplementary Table S4 online). Interestingly, each species had a very distinct set of positively co-occurring ASVs with only five ASVs shared between two species. Most of the co-occurring ASVs were assigned to micro-eukaryotes, with 18 (9%) assigned to Protozoa, and 24 (12%) to Chromista, or not assigned to any kingdom (58%; see Supplementary Table S3 online). These proportions reflect the assignments of the global dataset (see Supplementary Fig. S2 online).

### Correlation between amphipod abundance and eDNA metabarcoding diversity

**Figure 6:**
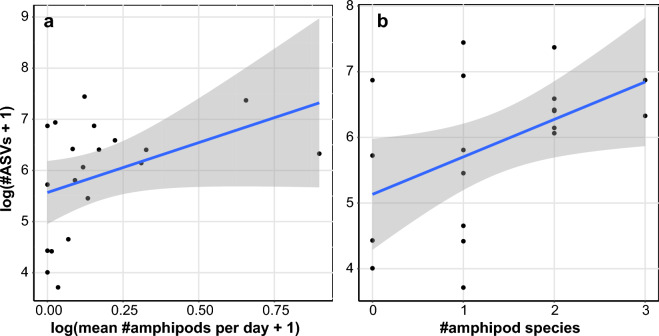
Correlation between molecular diversity and amphipods abundance or richness at each site. (**a**) Correlation between molecular diversity (as log number of ASVs) from the eDNA metabarcoding approach and the amphipod abundance from the citizen science approach (as log number of amphipods per day). The blue line shows the linear regression (y = 1.951x + 5.568) with the standard deviation in gray. (**b**) Correlation between molecular diversity (as log number of ASVs) from the eDNA metabarcoding approach and the amphipod richness from citizen science (as number of species). The blue line shows the linear regression (y = 0.571x + 5.133) with the standard deviation in gray.

To assess the relationship between amphipods and groundwater communities, we compared the number of amphipods collected by water providers at each site (log-transformation of the mean number of individuals collected per day) with the total diversity of ASVs from the eDNA metabarcoding dataset (log-transformation of the number of ASVs). Although the correlation is not significant (r = 0.407; *p* = 0.075), a high number of amphipod individuals is always associated to a high molecular diversity (Fig. 6a). The reverse, however, is not always true, with several sites exhibiting a high molecular diversity (number of ASVs), yet no or very few amphipods were found. When doing the same comparison with amphipod richness (number of species found at a site), the correlation is significant (r = 0.478; *p* = 0.033), and the observed pattern is similar (Fig. 6b). All sites exhibiting two or three amphipod species were associated with a high number of ASVs, whereas sites with no or only one amphipod species had a very variable molecular diversity, including both low and high values of ASVs.

## Discussion

As a proof-of-concept, we compared here two very different methods to overcome common challenges related to the study of groundwater amphipods, and possibly groundwater invertebrates in general. We showed that combining a citizen science approach based on the communal water providers’ participation with the use of a molecular approach (DNA barcoding) was particularly effective and provided in-depth knowledge on the distribution and abundance of amphipods in groundwater systems. While eDNA metabarcoding was somewhat less successful than citizen science with respect to the number of sites at which each species was detected, even when compared at the same sampling time, we identified aspects of the protocol that can be further improved. We also demonstrated that eDNA metabarcoding brought additional insights on the composition of groundwater communities, including microorganisms, and their associations to groundwater amphipods. Specifically, we found that different amphipod species are co-occurring with different micro-eukaryotic species, potentially indicating different habitat or food requirements. We also showed that amphipod abundance and diversity are correlated to the total richness (number of ASVs) at each site. These two results exemplify the potential of the two approaches tested in this study, opening new possibilities in the field of subterranean biology.

### Choosing the right approach according to one’s needs

We first implemented the citizen science approach in 2019^30^, with a pilot study deployed in the Swiss plateau. The results gave a proof-of-principle, showing the effectiveness of this approach to study groundwater amphipods. The question remained, however, how well the sampling reflected the amphipod diversity and distribution underground. Specifically, it was unclear if the scarcity of organisms collected (with often only one to very few individuals sampled despite high sampling efforts) was an indication of the actual low abundances of macro-invertebrates or if the sampling method was missing out local occurrences. Moreover, the citizen science approach does not allow the study of microorganisms, the main component of groundwater communities^27^.

When evaluating the efficiency of both methods in detecting groundwater amphipods, we can see that citizen science generates more species’ detections than eDNA, even when comparing samples collected at the same date (Fig. 2). When taking into account all citizen science samples, no detection of amphipod species was performed by eDNA only. This suggests that the amphipods collected by water providers are most likely representative of the populations living in groundwater. In fact, since both methods have possibly rather different biases, we would expect them to give different results if they were not exhaustive, as is often the case when comparing eDNA and traditional methods in other ecosystems (see e.g.,^31–33^). Instead, the detections from eDNA are included (i.e., nested) within the ones from citizen science, indicating that the latter is a good approach for studying groundwater organisms^30,34^.

Contrastingly, our results demonstrate that the eDNA metabarcoding protocol used in this study is not yet best suited to study groundwater amphipods. It systematically and accurately detects species with high abundances, but usually misses the rare ones (see Supplementary Fig. S1 online). These results are congruent with the growing amount of comparisons between eDNA-based and traditional approaches (^35^ and references therein), showing that eDNA is not as efficient to detect rare macroinvertebrates as traditional sampling. Several reasons have been suggested to explain this discrepancy, either related to the nature of eDNA (e.g., shedding rates, DNA degradation), or to technical difficulties (e.g., primer bias, sequencing depth) (e.g.,^36,37^). In our case, however, two main factors possibly explain the observed differences. First, the sampling effort was higher for the citizen science approach, even when considering samples collected at the same time point. All eDNA samples were composed of 40 L of water per site only, whereas thousands of liters of water passed through the filter nets during each week for the traditional sampling of whole individuals. Although there is no reason to think that both sample types, being so different, would require the same amount of water processed to give the same results, an increase in the volume of water filtered could potentially improve amphipod detections with eDNA.

Second, the low specificity of the primers used in this study had a major influence on the efficiency of eDNA metabarcoding. We modified existing primers designed to target arthropods^38^, so that they would amplify groundwater amphipods. Although an in silico test against all sequences present in GenBank’s database suggested that our primers should amplify mainly metazoans and particularly arthropods (see Supplementary Fig. S3 online), the reality is quite different and most of our sequences were either unassigned to any organisms or assigned to micro-eukaryotes (see Supplementary Fig. S2 online). Only 0.0002% of the reads corresponded to amphipod species, which led to the removal of relevant sequences during corrections for PCR errors or index-jump, as shown in Fig. 3. Moreover, the primers did not amplify *Crangonyx subterraneus*. Our protocol could thus be further improved by designing more specific primers for groundwater amphipods. This is, however, a very difficult task because of the high number of species described, especially for the genus *Niphargus*, and of their very high genetic divergence for COI^39^. Another solution would be to target another marker such as 16S or 18S for which other studies have successfully identified groundwater amphipods^25,33^. Since these markers are not traditionally used in phylogenetic studies of subterranean organisms in Europe, however, the number of references available for our target species is very low and more effort should thus be put into producing these references. Conversely, the use of a standard phylogenetic marker, such as a fragment of the nuclear 28S rRNA gene, is possible in terms of references available. In this case, however, the variability between species is rather low, and the fragment size required by sequencing technologies (< 400 bp) could lead to a loss in resolution, making it impossible to tell some species apart.

### Added-value of combining approaches: the best of both worlds

Despite targeting a small sampling area (443 km^2^), the amphipod gamma diversity detected with both approaches is relatively high for groundwater standards, and only very few regions in Switzerland such as the Jura mountains have a similarly high diversity^28,29^ (as well as unpublished data). Even in amphipods diversity hotspots, the richness of *Niphargus* per area is not much higher. For example, Borko et al.^4^ found that the majority of their 400 km^2^ grid cells studied in the Western Balkans contained less than 5 species, with a high turnover between grid cells. While we found 6 groundwater amphipods in the whole study region, the alpha diversity at each individual site, however, is systematically low with only one or two species found for most of them (Fig. 4a). This is surprising, as the distribution of the identified species is overlapping over the whole sampling range (Fig. 4b). A low diversity could be explained by three reasons. First, the abundance of the different species is so low that we were not able to sample the whole diversity at each site. Second, the requirements of the various species in terms of food or environmental conditions are different. Third, these amphipod species compete for resources and exclude each other at one site. Although we cannot completely exclude the first option, which is a recurring problem when studying groundwater organisms^2^, we showed earlier that the citizen science approach appears to give representative information. Moreover, our results suggest that the identified species have different requirements and might occupy different niches. Indeed, each species co-occurs with a very distinct set of ASVs from the metabarcoding dataset (Fig. 5, Supplementary Table S4 online). Since most of these ASVs are micro-eukaryotes, the co-occurrence pattern may be linked to trophic relationship (e.g., ASVs are a food source for amphipods, or they are parasites/epibionts of amphipods). They could also have no relationship, and only require similar environmental conditions. As our dataset is limited to 20 sites, this hypothesis should be further tested on a larger scale.

Similarly, we demonstrated that the abundance and diversity of amphipods are tightly linked to the overall groundwater diversity (Fig. 6). This correlation could be due to environmental conditions (e.g., different amount of oxygen, presence of pollutants) but could also result from hostile physical properties (e.g., reduced pore size). It could also indicate that amphipods can only thrive when the conditions are favorable and the overall diversity is abundant. Since the organisms living in groundwater provide important ecosystem services such as improving water quality^9^, evaluating the state of groundwater diversity should be an important aspect of aquifer monitoring, and this could be done by sampling amphipods. It is important to note, however, that their absence does not necessarily imply a low diversity. Indeed, we found in some sites a high number of ASVs without the presence of amphipods. Although sampling bias cannot be completely excluded, this pattern could result from the physical parameter of the aquifer at those sites, with potentially small pore sizes that would only support the development of microorganisms. Given the small scale of our study, our results should be expanded by including more diverse locations. Nonetheless, we showed here that the combination of citizen science and eDNA metabarcoding is a suitable tool for gaining insights into the ecology of groundwater organisms.

## Conclusion

In the light of our results, both tested methods have their pros and cons, and have complementing advantages with respect to different questions and needs. On one hand, taking water samples for eDNA metabarcoding is faster, less work-intensive to process, and is thus easier to scale up spatially (i.e., sample at the regional or national level) and temporally (e.g., yearly monitoring). Moreover, it gives information on the whole community, and not only a restricted set of species of interest. However, at the current state of our study, it seems less effective in detecting specific macroinvertebrates, such as amphipods, and does not provide phenotypic information. On the other hand, citizen science is very effective for the study of groundwater amphipods, and potentially other macro-invertebrates. It allows performing morphological studies, species descriptions, but could also be used to investigate population genetics patterns or trophic interactions (through stable isotopes or fatty acids analyses for example). It is also engaging for stakeholders and practitioners to get them invested in protecting groundwater biodiversity. However, the execution of the citizen science approach and the repeated sampling using nets and sampling of individuals is relatively time-intensive, and requires close mentoring of people involved. While it can be scaled up geographically, it cannot be repeated too often if we want to keep the parties involved interested in the project, precluding temporal upscaling. We thus suggest here two new approaches to study groundwater organisms that can be used separately, or even better in combination, and that can help tackling unexplored questions in subterranean biology.

## Material and methods

### Study sites

Groundwater represents 80% of the drinking water supply in Switzerland^40^. The method for collecting and using this water is quite specific to Switzerland and surrounding alpine areas. Specifically, water is passively collected from horizontal pipes accessing it from a shallow aquifer and leading it into spring catchment boxes (SCBs; Fig. 1b). Water provision is managed by municipalities. By contacting them, we established a citizen science approach^30^, where all sampling performed for this study was done in collaboration with water providers. We conducted our study in the 443 km^2^ catchment basin of the river Töss in North-Eastern Switzerland (Fig. 1a). The 20 sampled sites are located in the Swiss Molasse Basin mainly consisting of freshwater and marine alluvial deposits, and composed of sandstones, silt and marls. The amphipods in this region are well-studied^28^, with a particular focus on groundwater species^14,34^. All 20 sampled sites are evenly distributed across the study area, and belong to 20 different municipalities. Two types of samples were collected at each site: (1) water providers collected whole organisms using filter nets, and stored them for subsequent identification via molecular barcoding (hereafter referred to as citizen science), and (2) we took water samples for environmental DNA metabarcoding (hereafter referred to as eDNA).

### Citizen science

#### Sampling

We used a previously established citizen science approach to collect the amphipods, following the methods described in Alther et al.^30^ and Alther et al.^41^. The collection of organisms was performed by the water providers between March and July 2021, following a standardized procedure. All participants received a sampling kit containing the necessary material and instructions to perform the sampling in a similar manner. First, they collected organisms present in the overflow basin of their SCB using the provided aquarium net (mesh size 0.35 mm; Fig. 1b item 1). Then, they attached a bag net (mesh size 0.8 mm) to all the outflow pipes available in the SCB (Fig. 1b item 2). The nets were controlled every week for several consecutive weeks (up to 10), and organisms present in the nets were transferred into 5 mL tubes filled with 80% molecular grade ethanol. Water providers returned their samples to us within days after the sampling date and we stored them at 4 °C until further processing.

#### DNA extraction, amplification, and sequencing

Upon arrival in our lab, samples were manually checked and any amphipod organism present in a sample was isolated in individual tubes filled with fresh 80% molecular grade ethanol. DNA extraction was performed in 96-well plates using the Chelex^®^ 100 protocol described by Walsh, et al.^42^. We used either three legs (pereopods 5 to 7) as tissue source for big individuals, or half of the organisms for small or damaged individuals. The tissue parts were incubated overnight at 55 °C in a lysis solution composed of 150 µL of 10% Chelex^®^ 100 biotechnology grade resin solution (Bio-Rad Laboratories, Inc) and 10 µL of proteinase K (20 mg.mL^−1^; VWR International, LLC). We then transferred 40 µL of supernatant into a new plate and stored this extract at − 20 °C.

We amplified a 658-bp COI fragment using modified versions of the Folmer primers by Astrin and Stüben^43^ and Astrin and Stüben^44^, as follows: LCO1490-JJ 5’-CHACWAAYCATAAAGATATYGG-3’ and HCO2198-JJ 5’-AWACTTCVGGRTGVCCAAARAATCA-3’. We performed the PCR in a volume of 25 µL consisting of 1 X Master Mix from the Qiagen® Multiplex PCR Kit, 0.5 X Q-solution, 0.5 µM of each primer and 2 µL of undiluted template DNA. The PCR was performed on Biometra T1 Thermocyclers (Analytik Jena GmbH, Germany), and started by a denaturation step at 95 °C for 15 min, followed by 35 cycles of 94 °C for 30 s, 52 °C for 90 s and 72 °C for 60 s, and ended by an extension step at 72 °C for 10 min. We checked the amplification success with a QiAxcel® Screening Cartridge (Qiagen^®^, Germany). PCR products were then purified using the ExoSAP-IT™ PCR product cleanup kit (Thermo Fisher Scientific, Inc.) following the manufacturer’s instructions. Purified PCR products were sent to Microsynth AG, (Switzerland) for Sanger sequencing on both directions.

#### Taxonomic assignment

The forward and reverse sequences for each individual were manually checked and aligned using CodonCode© Aligner v-10.0.1. Only sequences with a sufficient quality on more than 400 bp were considered for taxonomic assignment. We compared our sequences to a lab-internal database using the package blaster, implementing a blast®-like algorithm^45^. We assigned each sequence to a species if the query coverage was at least 99% and the percent identity above 94%. This threshold is based on a barcoding gap analysis performed with all the sequences available in our internal database (see Supplementary Fig. S4 online). If no reference from the lab-internal database matched with our query sequence, we compared it to BOLD^46^, using their API interface in R. If both approaches failed we used blast®^47^ to align the sequences against NCBI’s GenBank nt database^48^.

### Environmental DNA

#### Sampling

The eDNA sampling was performed between April and June 2021, always at one of the dates where the filter nets were checked for amphipods according to the citizen science protocol. We collected 4 × 10 L of water in two 20 L plastic canisters at each site in less than 5 min (Fig. 1b item 3), and we brought them back to the laboratory in cooling boxes within two hours for subsequent filtration. The water was filtered through enclosed Sterivex™ filter units (pore size of 0.22 µm) using a peristaltic pump at a flow rate of approximately 6.7 mL s^−1^. The four replicates per site (10 L per filter) were then stored at − 20 °C until DNA extraction.

To avoid contamination between sites, most of the material used was disposable and sterile. We only reused the canisters and tubing for the pump, which were cleaned between sites by soaking them into a bleach solution (2 L of commercial < 2% hypochlorite solution diluted with 3 L of molecular grade water) for at least 30 min. We subsequently rinsed them three times with molecular grade water. We wore gloves at all sampling steps to avoid external contamination, including material preparation. Finally, to check for potential contamination despite our preventive measures, we performed filtration controls by collecting molecular grade water in the canisters following the decontamination protocol detailed above. Three controls were produced with 10 L of this water filtered for each, following the same protocol as the other samples.

#### DNA extraction and library construction

To reduce the risk of external DNA contamination, all DNA extractions and the first PCR step were performed in a clean-lab with constant air overpressure, and in the absence of PCR products. We used the DNeasy^®^ PowerWater^®^ Sterivex™ Kit (Qiagen^®^, Germany) with a modified version of the manufacturer’s protocol where the bead-beating steps (step 12 and 13) are omitted. We pre-heated the elution buffer at 70 °C before passing the same buffer twice on each column to increase the yield without decreasing the final concentration. Three extraction controls were produced by adding 0.9 mL of lysis buffer (ST1) to a clean filter unit. We further processed them following the same protocol as the other samples. We diluted all the samples 1:10 of their original concentration to decrease the presence of potential PCR inhibitors and ensure a proper amplification.

Amphipod species from the genus *Niphargus* are the main groundwater species found in Switzerland^28,30^. Since their COI fragment is not well amplified by metabarcoding primers traditionally used to target macroinvertebrates^49,50^, we modified those developed by Vamos, et al.^38^, amplifying a short fragment of 205 bp, as follows:

fwhF2_Niph 5′-GGRTGAACAGTWTAYCCTCC-3′ and

fwhR2n_Niph 5′-GTRATWGCTCCWGCTARMACTGG-3′. We tested their ability to amplify *Niphargus* amphipods by using available DNA in our lab from specimens of 48 different species.

We prepared the library using a two-step PCR approach. All details of the protocol are presented in Couton, et al.^27^. Briefly, we performed the first amplification step for each filter replicate separately. A total of 15 PCR replicates were carried out for each filter. We performed them by groups of three, and each of the five groups was identified with unique 8-bp tags. Two amplification controls were produced at this step. After the first PCR, all tagged PCR replicates from a same filter were pooled and we carried out a second PCR to bind the Nextera^®^ index adapters (Set A) to the fragments. Finally, we pooled all samples, including all controls, at equimolar concentration. The library was sequenced (paired-end, 250 cycles) on an Illumina MiSeq platform (Illumina, Inc., USA) at the Genomic Diversity Center, ETH Zurich, Switzerland.

#### Read processing

Each sample was demultiplexed first on its index combination by the sequencer, returning one file per filter replicate. We performed a second demultiplexing on the tags to disentangle PCR replicates using cutadapt v-2.8^51^. At the same time, both primers and tags were removed from the sequences. Then, we used dada2 v-1.13.1^52^ to produce a set of amplicon sequence variants (ASVs). At the demultiplexing step, reads can be attributed to the wrong sample/replicate, a phenomenon called “index-jump”^53^. We thus added eight unused index combinations in the demultiplexing sheet in order to assess the proportion of reads falsely attributed to these non-existing samples. For each ASV, we calculated the proportion of reads assigned to each of the index combinations, as compared to the abundance of this ASV in the whole dataset. The maximum value of these observed proportions within an unused index combination was used as a threshold for index-jump correction. We thus removed, in each sample, any ASV for which the proportion did not account for more than the chosen threshold. Additionally, we removed potential PCR errors by removing ASVs only present in one out of the 20 replicates per site (four filters x five PCRs). We processed the eight negative controls (three sampling controls, three extraction controls, and two PCR controls) with the same protocol and none of them contained any reads after correction. Detailed scripts for each of the tools used in this pipeline are available online (https://github.com/joarwrie/NiphComp).

#### Taxonomic assignment

We aligned all ASVs remaining after correction against our lab-internal database containing COI sequences for all amphipod species (surface and groundwater) present in Switzerland^29^, and including the sequences produced by the citizen science approach. This database contains at least one specimen for each species that was identified both morphologically and molecularly. We used the blast^®^ command line tool^47^ with default values, returning a maximum of 500 hits per query. After removing alignments not covering at least 99% of the query, we considered only those with more than 94% identity. As previously described for the citizen science part, this threshold was chosen based on a barcoding gap analysis (see Supplementary Fig. S1 online). Each ASV was assigned to the species with a reference having the highest identity.

For all ASVs not assigned to an amphipod species, we performed a second taxonomic assignment using the Dark mAtteR iNvestigator (DARN) tool^54^. This pipeline is based on a phylogenetic placement algorithm and returns different placement possibilities for each ASV with their likelihood. Our objective was only to identify what type of organisms could be associated with our unknown sequences so we considered only high taxonomic level assignments (kingdom and phylum). We associated each ASV to the taxon with the highest maximum likelihood weight ratio (LWR), and we only kept this assignment if the LWR was greater than 0.5. If no placement had a LWR greater than 0.5 for a target ASV, we classified it as “unassigned” (see Supplementary Fig. S2 online).

### Statistical analyses

We compared the citizen science and eDNA metabarcoding datasets on their ability to detect groundwater amphipod species at each site. For these comparisons, the abundance of amphipod individuals collected at each site was corrected to account for the difference in sampling effort. Since the sampling duration was comprised between 7 and 71 days, we divided the total number of individuals at each site by the number of days this site was sampled, thus giving a mean number of amphipods per day.

The Pearson correlation coefficient between amphipod abundance identified with citizen science and the number of ASVs at each site was computed using the *cor.test* function of the stats R package^55^. We log-transformed both variables prior to computation. The same coefficient was computed between the amphipod diversity identified with citizen science and the number of ASVs at each site. In this case, we only log-transformed the number of ASVs.

In order to identify ASVs potentially co-occurring with each of the amphipod species identified within the citizen science dataset, we compared their presence/absence for all sites with the distribution of each ASV occurring at more than one site. We used the R package cooccur^56^ where co-occurring probabilities are calculated using a hypergeometric distribution. All analyses were performed in R v-4.1.3^55^.

### Supplementary Information

Supplementary Information 1.

Supplementary Information 2.

## Data Availability

Raw sequence reads are deposited on NCBI, in the SRA: BioProject PRJNA905821. All new barcoding sequences produced for this publication are deposited in GenBank under the accession numbers OR608123-OR608190. The metabarcoding and analyses scripts used in this study are available at https://github.com/joarwrie/NiphComp.
