# Supplementary material for: Integrating citizen science and environmental DNA metabarcoding to study biodiversity of groundwater amphipods in Switzerland
Source: Sci Rep. 2023 Oct 23;13:18097. doi: 10.1038/s41598-023-44908-8 (PMC10593815; doi:10.1038/s41598-023-44908-8)
Supplement: Supplementary file 1 — Supplementary Information 1. [file 41598_2023_44908_MOESM1_ESM.docx]

**Supplemental Information for:**

**Integrating citizen science and environmental DNA metabarcoding to study biodiversity of groundwater amphipods in Switzerland.**

Marjorie Couton^1,^*, Angela Studer^1^, Samuel Hürlemann^1^, Nadine Locher^1^, Mara Knüsel^1,2^, Roman Alther^1,2^, Florian Altermatt^1,2,^*

^1^ Eawag: Swiss Federal Institute of Aquatic Science and Technology, Department of Aquatic Ecology, Überlandstrasse 133, CH-8600 Dübendorf, Switzerland.

^2^ Department of Evolutionary Biology and Environmental Studies, University of Zurich, Winterthurerstrasse 190, CH-8057 Zürich, Switzerland.

* Corresponding authors: marjorie.couton@eawag.ch and florian.altermatt@eawag.ch

**Figure S1** Abundances of the different amphipod species at each site, in mean number of individuals per day for the citizen science approach and in number of reads for the eDNA metabarcoding approach. **a)** Comparison of the two approaches when considering only citizen science samples collected at the same date as eDNA sampling. **b)** Comparison of the two approaches when considering all citizen science samples collected for this study.


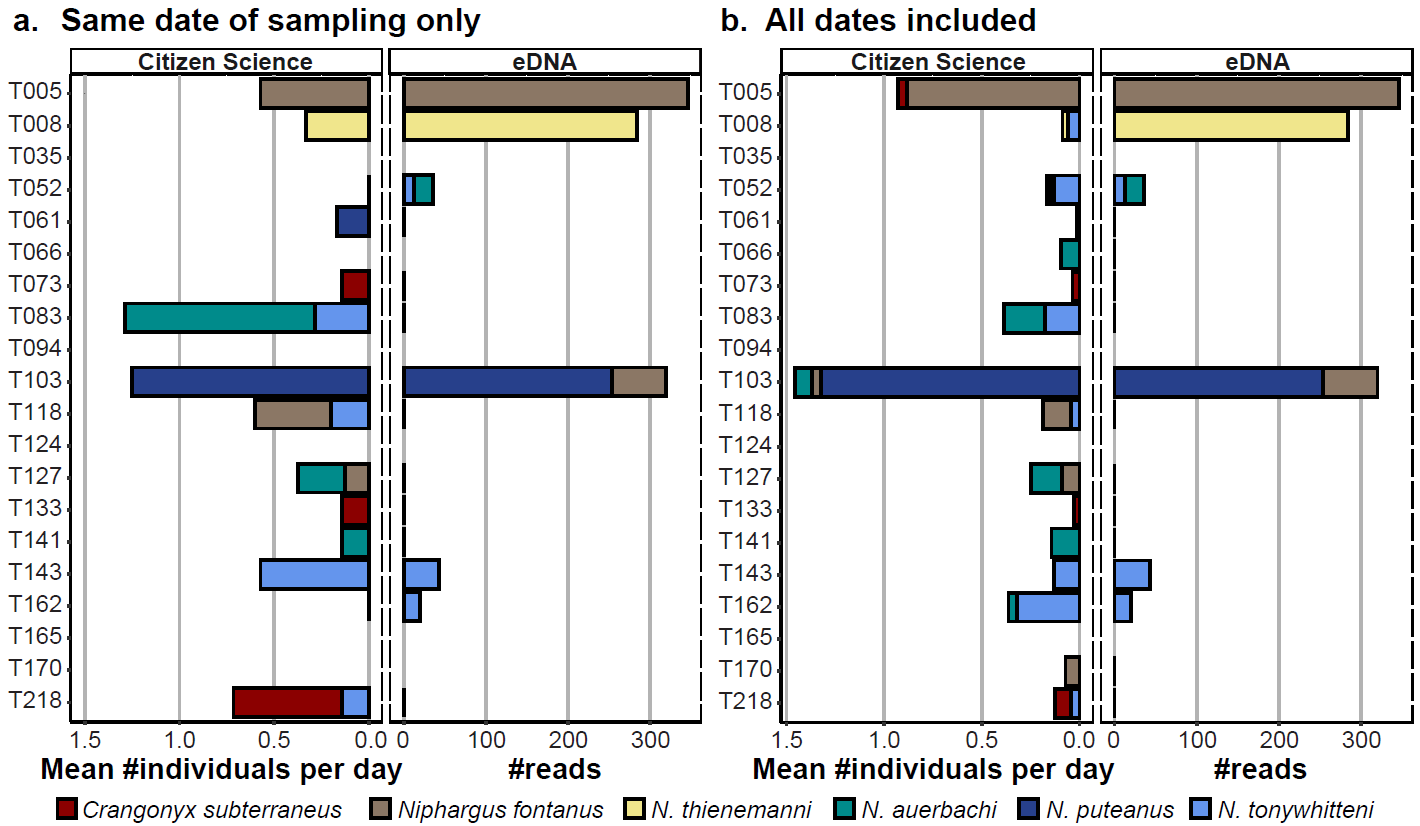


**Figure S2** Distribution of read assignments at the kingdom level and for the different

metazoan phyla observed, using a phylogenetic placement approach.


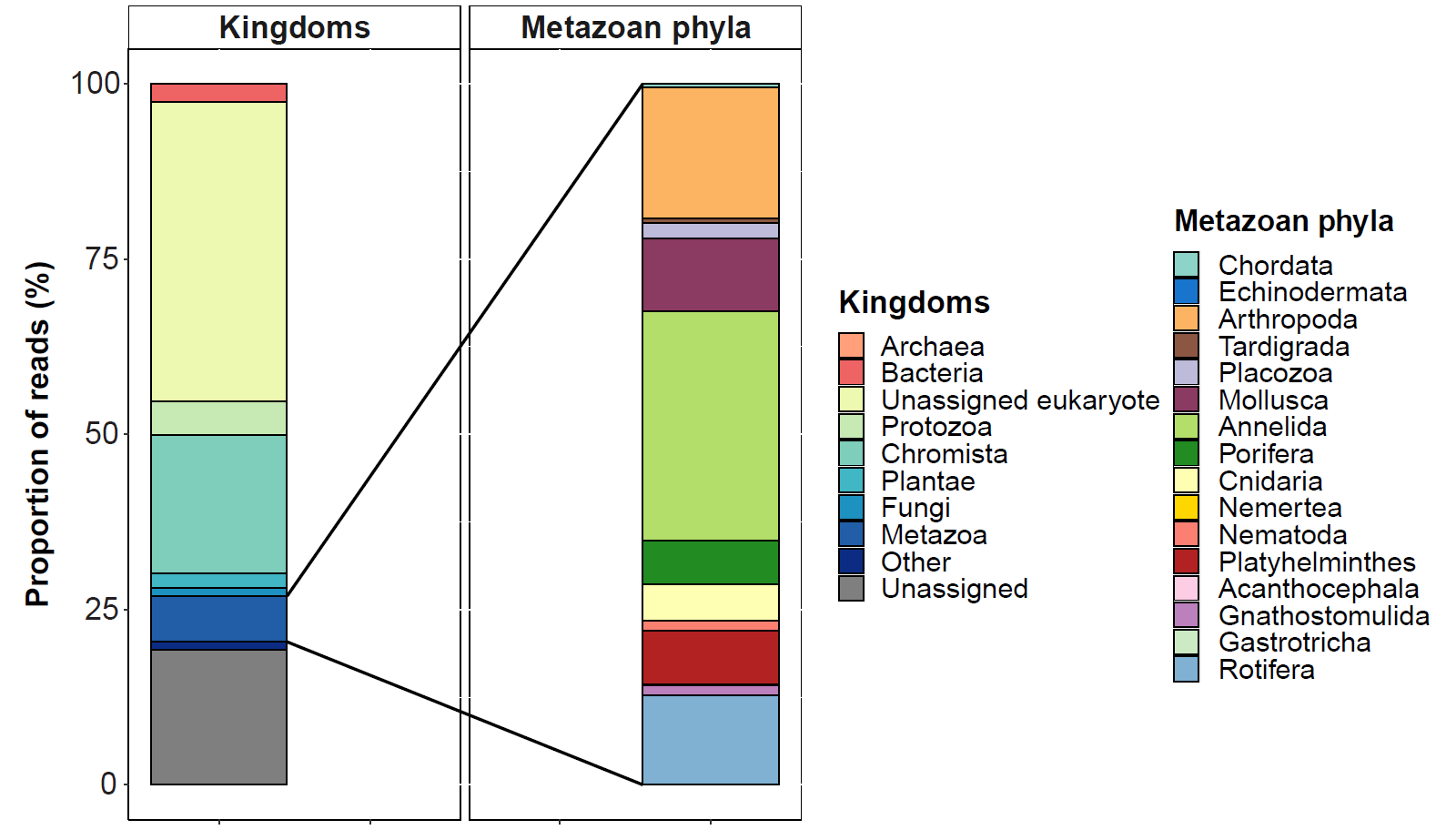


**Figure S3** Proportion of taxa amplified by the modified primers fwhF2_Niph/fwhR2n_Niph according to an *in silico* PCR using ecoPCR against GenBank nt database for each kingdom (**A**) or each metazoan phylum (**B**). Only four mismatches were allowed per primer with no mismatch possible on the two last bases at the 3’ end. Only the amplified sequences between 200 and 210 bp were considered.

**
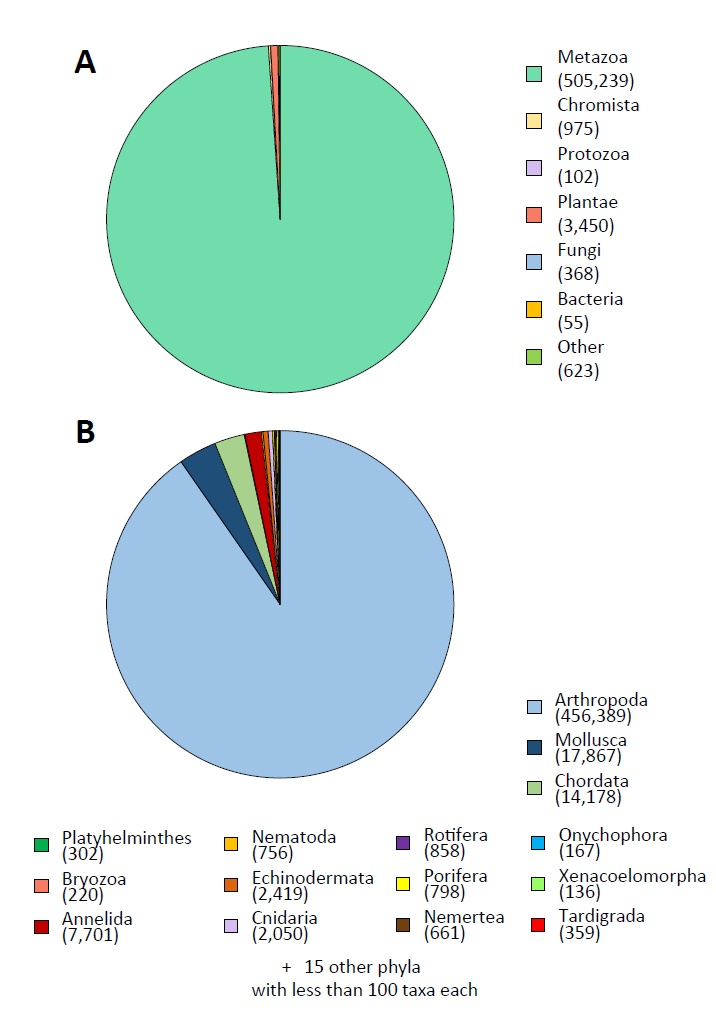
**

**Figure S4** Density plot displaying the similarity (in % identity) between two sequences of the same species (blue), genus (yellow) or family (red) within groundwater amphipod species. The results were obtained by collecting 1059 sequences from GenBank belonging to 127 species from the genera *Niphargus* and *Crangonyx*. All sequences were compared in a pairwise approach using the global alignment tool needle with default parameters. The magenta line represent the chosen threshold (94%) to assign a sequence to a species in our study.

**
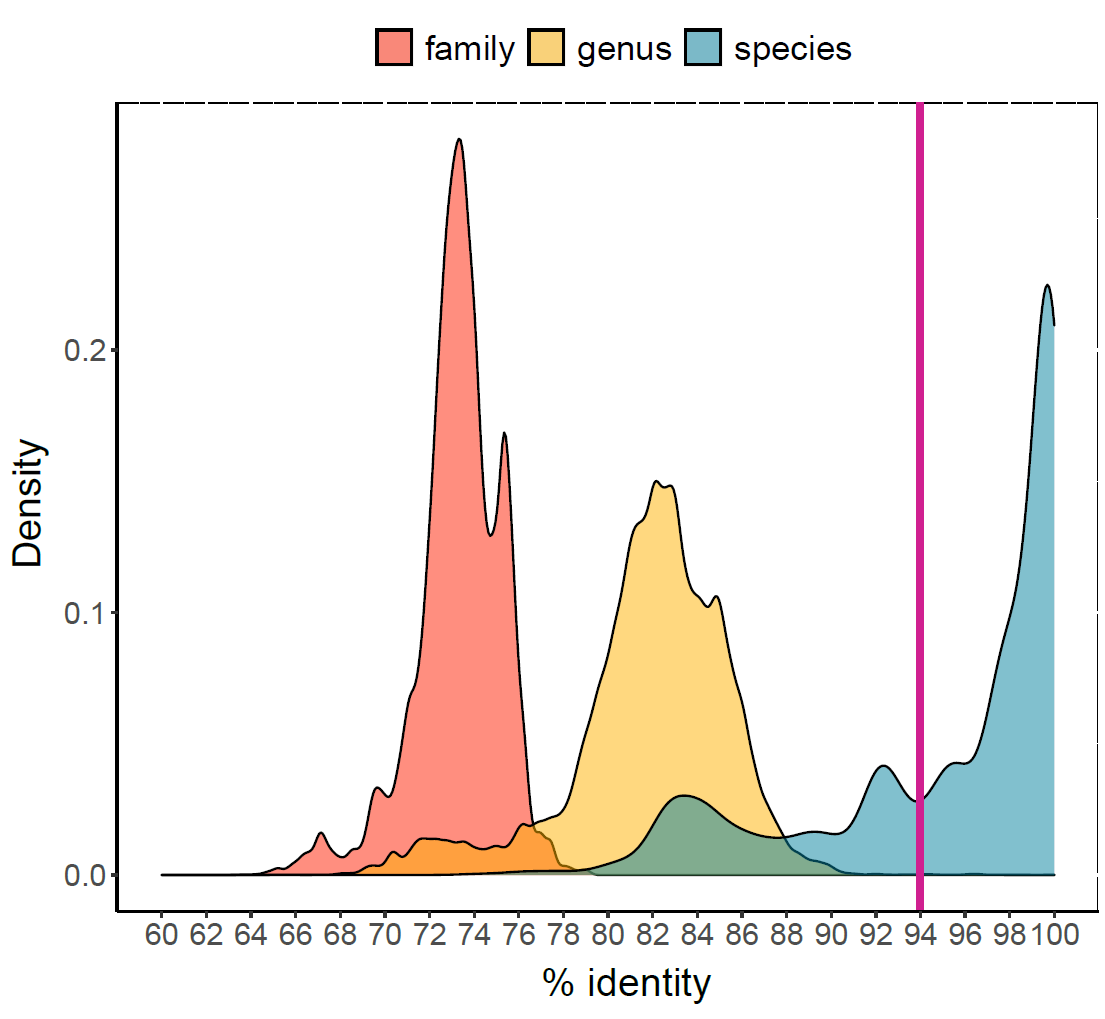
**
